# Evaluation of high throughput gene expression platforms using a genomic biomarker signature for prediction of skin sensitization

**DOI:** 10.1186/1471-2164-15-379

**Published:** 2014-05-16

**Authors:** Andy Forreryd, Henrik Johansson, Ann-Sofie Albrekt, Malin Lindstedt

**Affiliations:** Department of Immunotechnology, Lund University Medicon, Village 406, Scheelevägen 2, 223 81 Lund, Sweden

**Keywords:** Genomic biomarker signature, GARD, Microarrays, nCounter®, BioMark HD™, OpenArray®

## Abstract

**Background:**

Allergic contact dermatitis (ACD) develops upon exposure to certain chemical compounds termed skin sensitizers. To reduce the occurrence of skin sensitizers, chemicals are regularly screened for their capacity to induce sensitization. The recently developed Genomic Allergen Rapid Detection (GARD) assay is an *in vitro* alternative to animal testing for identification of skin sensitizers, classifying chemicals by evaluating transcriptional levels of a genomic biomarker signature. During assay development and biomarker identification, genome-wide expression analysis was applied using microarrays covering approximately 30,000 transcripts. However, the microarray platform suffers from drawbacks in terms of low sample throughput, high cost per sample and time consuming protocols and is a limiting factor for adaption of GARD into a routine assay for screening of potential sensitizers. With the purpose to simplify assay procedures, improve technical parameters and increase sample throughput, we assessed the performance of three high throughput gene expression platforms - nCounter®, BioMark HD™ and OpenArray® - and correlated their performance metrics against our previously generated microarray data. We measured the levels of 30 transcripts from the GARD biomarker signature across 48 samples. Detection sensitivity, reproducibility, correlations and overall structure of gene expression measurements were compared across platforms.

**Results:**

Gene expression data from all of the evaluated platforms could be used to classify most of the sensitizers from non-sensitizers in the GARD assay. Results also showed high data quality and acceptable reproducibility for all platforms but only medium to poor correlations of expression measurements across platforms. In addition, evaluated platforms were superior to the microarray platform in terms of cost efficiency, simplicity of protocols and sample throughput.

**Conclusions:**

We evaluated the performance of three non-array based platforms using a limited set of transcripts from the GARD biomarker signature. We demonstrated that it was possible to achieve acceptable discriminatory power in terms of separation between sensitizers and non-sensitizers in the GARD assay while reducing assay costs, simplify assay procedures and increase sample throughput by using an alternative platform, providing a first step towards the goal to prepare GARD for formal validation and adaption of the assay for industrial screening of potential sensitizers.

## Background

Allergic contact dermatitis (ACD) is characterized by redness of the skin, accompanied by periodic and recurrent episodes of itching [1]. The disease is a type IV delayed type hypersensitivity reaction initiated and mediated by cytotoxic CD8+ T cells and CD4+ Th1 cells [2] and develops upon repeated or prolonged exposure to contact allergens [3]. With a prevalence of 15-20% [4, 5], ACD has rapidly become a major occupational and environmental health problem [6, 7]. ACD is a chronic condition and the recommendation to affected individuals is complete avoidance of inducing contact allergen [3].

Contact allergens capable of inducing ACD are termed skin sensitizers. An important group of skin sensitizers are chemical compounds present in common household products such as cosmetics and personal care products. To limit and control the use of sensitizers in such products, chemicals are regularly screened for their capacity to induce sensitization. Historically, such assays have relied exclusively on animal experimentation, with the murine local lymph node assay (LLNA) [8] being the preferred method. However, as of March 2013, an EU legislation [9] imposed a ban on the use of animals for safety assessment of cosmetic products, regardless of the availability of non-animal test as an alternative to animal testing. Thus, the demand for alternative *in vitro* methods for prediction of chemical sensitization is urgent.

Recently, we developed the Genomic Allergen Rapid Detection (GARD) assay, a novel alternative to animal testing for identification and risk assessment of human skin sensitizing chemicals using a predictive genomic biomarker signature termed the GARD prediction signature (GPS) [10]. The GPS was established using a panel of reference chemicals comprising 18 well known sensitizers and 20 non-sensitizers. By stimulating the myeloid cell line MUTZ-3 with the panel of reference chemicals, we were able to identify the 200 most potent discriminatory transcripts between non-sensitizers and sensitizers. The information given by differentially expressed transcripts in the GPS was used to train a support vector machine model (SVM) [11]. Subsequently, the SVM model is used for classification of unknown chemical compounds as either sensitizers or non-sensitizers.

The technical format currently used for transcriptional analysis in the GARD assay is Affymetrix® whole-transcriptome microarrays. The array platform has been valuable during assay development to identify differentially regulated genes in the GPS from the entire transcriptome, yet it suffers from drawbacks in terms of low sample throughput, high cost per sample and time consuming protocols. Furthermore, the microarray platform has known limitations in terms of sensitivity, reproducibility and dynamic range [12–15]. For an *in vitro* method to be used for regulatory purposes and eventually for industrial screening of sensitizers, it needs to be validated according to internationally recognized procedures [16]. With the purpose to prepare GARD for formal validation under the supervision of The European Union Reference Laboratory for alternatives to animal testing (EURL ECVAM), and in order to simplify assay procedures, improve technical parameters and increase sample throughput, the GARD assay would benefit from a technical platform transfer. The number of available technologies for high throughput quantitative transcriptome analysis is steadily increasing, but only a handful of comparisons between gene expressions measurements on various platforms exist. Thus, there is a need to evaluate and compare results obtained from the various technologies to assess reliability and biological significance of measurements. We performed a systematic head to head comparison to assess the performance of three of the most prominent high throughput gene expression platforms, including the nCounter® analysis system (NanoString® technologies) [17], BioMark HD™ system (Fluidigm® corporation) [18] and the OpenArray® system (Life Technologies™) [19]. The nCounter® analysis system is a novel hybridization based technology enabling gene expression measurements to be performed directly from cell lysate. The technology use transcript specific color coded molecular probe pairs to capture and count individual transcripts and has several advantages in comparison to microarray platform including easy protocols, less hands-on time and higher sample throughput [17]. BioMark HD™ and OpenArray® are RT-qPCR based technologies which is considered as the gold standard for quantitative transcription analysis due to its high sensitivity, high reproducibility and large dynamic range [19–21]. RT-qPCR is however a low throughput technology in its conventional format. Utilizing slightly different strategies, both BioMark HD™ and the OpenArray® system provides technologies that aims to conserve the intrinsic analytical benefits of the conventional RT-qPCR while enabling gene expression measurement in a high throughput format [22]. Both systems use streamlined protocols that enable progression from cDNA into results in less than three hours for the OpenArray® system and less than seven hours for the BioMark HD™ system. In this report, we present a per application relevant comparison and evaluation of the nCounter®, BioMark HD™ and the OpenArray® platforms in the context of the GARD assay. This report is not intended to serve as a general recommendation of a particular platform, but rather to investigate the trade-offs in terms of price, throughput, protocols, analytical performance and applicability of a selection of high throughput platforms for the GARD assay. Primary focuses were to compare non-technical parameters such as sample throughput, price per sample and simplicity of protocols between the platforms as well as to evaluate the more technical parameters in terms of precision and data consistency within each platform, to assess inter-platform consistency, and to determine if transcriptional analysis performed on the evaluated platforms could be applied to the GARD model to classify sensitizers from non-sensitizers. In conclusion, we found that it was possible to achieve acceptable detection sensitivity, reproducibility and discriminatory power of the GARD assay while at the same time simplify assay procedures and reduce assay cost when using any of the suggested high-throughput platforms evaluated in this study.

## Results

### Biological model for evaluation of high throughput platforms and phenotypic analysis of unstimulated MUTZ-3 cells

The GARD assay was chosen as a biological model to achieve a per application relevant assessment of the performance of selected high throughput platforms. The cell line used in the GARD assay is the dendritic cell (DC)-like human myeloid leukemia-derived cell line MUTZ-3 [23]. Analogous to primary DCs, MUTZ-3 cells express CD1a, HLA-DR, CD54, CD80 and CD86. The MUTZ-3 population also consists of subpopulations of CD14+, CD34+ and double negative cells as previously described [24]. Phenotypic analysis of cells was performed prior to each round of stimulation to ensure that MUTZ-3 cells were in an immature stage. Cell surface expression levels of the markers CD54, CD86, CD80, HLA-DR, CD14, CD34 and CD1a were verified using flow cytometry prior to stimulations. Results are illustrated in Table 1 and correlated with previously published examples of phenotypic profiles for the MUTZ-3 cells [10].

**Table 1:** **Phenotype of MUTZ-3 cells**

Cell surface marker	% positive cells	Standard deviation
**CD1a**	28.4	2.1
**CD14**	6.1	0.9
**CD34**	57.0	6.2
**CD54**	72.1	1.8
**CD80**	0.4	0.2
**CD86**	27.8	1.8
**HLA-DR**	87.4	4.1

Expression levels of the cell surface markers CD54,CD80,CD86,HLA-DR,CD1a, CD14 and CD34 were determined using flow cytometry to ensure proliferating cells were not differentiated. Results illustrates average percentage of positive cells together with standard deviation (n = 3).

### Inducible up-regulation of CD86 cell surface marker in response to stimulation of MUTZ-3 with skin sensitizers

**Figure 1:**
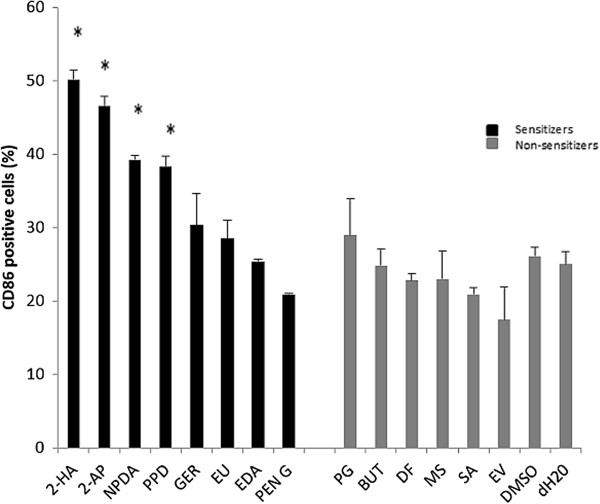
**Cell surface expression of CD86 in MUTZ-3 cells after stimulation with sensitizing and non-sensitizing chemicals.** Levels of expression of the cell surface marker CD86 were determined using flow cytometry after stimulation with various chemicals for 24 h. Figure illustrates percentage of CD86 positive cells for each chemical (n = 3). Gating were performed to exclude dead cells/debris and quadrants were established by comparing with isotype controls. Statistics were performed using Student’s t-test (*p < 0.05).

To enable a comprehensive evaluation between platforms, a panel of 16 chemical compounds including eight sensitizers and eight non-sensitizers initially being used to define the GARD prediction signature (GPS) were prepared for stimulation of MUTZ-3 cells (Table 2). Each stimulation was performed in biological triplicates, generating a dataset comprising 48 chemical stimulations. The GARD assay uses the inducible cell surface expression of CD86 after chemical stimulation as a general measure of the maturity state of the cells and as a quality control to ensure bioavailability of chemical stimulations. Cell surface expression of CD86 was confirmed after 24 h of stimulation with chemicals using flow cytometry (Figure 1). CD86 was significantly up-regulated on cells stimulated with the sensitizers 2-hydroxyethyl acrylate, 2-aminophenol, 2-nitro-1,4-phenylendiamine and p-phenylendiamine in comparison to vehicle controls. None of the non-sensitizers induced a significant up-regulation of CD86. The outcome of the experiment correlated with previously published data [10].

**Table 2:** **List of chemical compounds used during evaluation of platforms**

Compound	Abbrev.	Potency according to LLNA	Vehicle	GARD input concentration (μM)
***Sensitizers***				
2-Aminophenol	2-AP	Strong	DMSO	100
2-nitro-1,4-phenylenediamine	NPDA	Strong	DMSO	300
p-Phenylenediamine	PPD	Strong	DMSO	75
Ethylenediamine	EDA	Moderate	Water	500
2-hydroxyethyl acrylate	2-HA	Moderate	Water	100
Eugenol	EUG	Weak	DMSO	300
Geraniol	GER	Weak	DMSO	500
Penicillin G	PEN G	Weak	Water	500
***Non-sensitizers***				
1-Butanol	BUT		DMSO	500
Dimethyl formamide	DF		Water	500
Ethyl vanillin	EV		DMSO	500
Metyl salicylate	MS		DMSO	500
Propylene glycol	PG		Water	500
Salicylic acid	SA		DMSO	500
***Vehicle controls***				
Water	dH_2_O		-	-
Dimethyl sulfoxide	DMSO		-	-

List of chemical compounds used for evaluation of platforms. List includes information on sensitizing potency, vehicle, GARD input concentration and abbreviation for each chemical.

### Transcriptional profiling of MUTZ-3 using high throughput gene expression platforms

**Figure 2:**
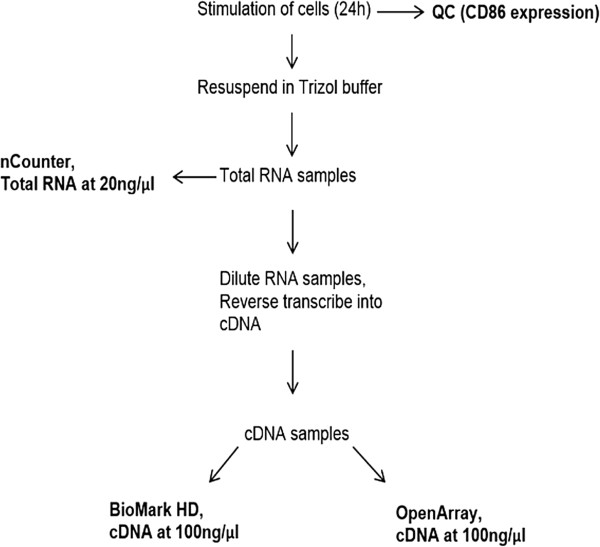
**Experimental workflow for evaluation of high throughput platforms.** Schematic view of the experimental design illustrating the procedure from chemical stimulation to mRNA quantification on the various platforms.

Selection of transcripts for this study was performed in two consecutive steps. In a first step, transcripts were selected in order to obtain a representative expression profile from the GPS to allow for a decisive evaluation of technical parameters such as intra platform reproducibility and limit of detection. In this step, transcripts were selected to cover the whole dynamic range of gene expression present in the original GPS (including low, medium and highly expressed transcripts) as well as to include heavily regulated transcripts, weakly regulated transcripts and to include both up- and down regulated transcripts when comparing expression profiles of chemically stimulated cells to non-stimulated cells. For the transcripts selected in the first step, a second round of selection was performed to include only the most potent predictor genes to allow for assessment of the biological relevance of gene expression data in terms of the GARD assay. Selection of the most potent predictor genes were based on validation call frequencies as described in [10]. The abundance of each transcript included in this study (Table 3) was determined on all platforms using samples from the same cellular stimulation. Data were analyzed to evaluate intra-platform reproducibility, inter-platform reliability and relevance of data produced on each platform for construction of a predictive model for skin sensitization in the GARD assay. A schematic view of the experimental workflow from chemical stimulation to mRNA quantification using the various platforms is illustrated in Figure 2. The data from the high throughput platforms were compared to Affymetrix® microarray data from a previous study [10]. A comparison of non-technical parameters of evaluated platforms is illustrated in Table 4.

**Table 3:** **List of transcripts used during evaluation of platforms**

Gene symbol	Entrez gene ID	DELTAGene™ assay ID	NanoString® probe ID	Life technologies™ assay ID
*ADAM20*	8748	GEP00055625	NM_003814.4:1420	Hs01083178_s1
*CD33*	945	GEP00055628	NM_001177608.1:730	Hs00233544_m1
*CD86*	942	GEP00055621	NM_175862.3:1265	Hs01567026_m1
*CD93*	22918	GEA00016079	NM_012072.3:4270	Hs00362607_m1
*DHCR24*	1718	GEA00025118	NM_014762.2:975	Hs00207388_m1
*DHX33*	56919	GEP00055629	NM_001199699.1:2873	Hs01063767_m1
*FAS*	355	GEP00055622	NM_152876.1:1740	Hs00907759_m1
*FASN*	2194	GEA00032375	NM_004104.4:5387	Hs00188012_m1
*FDXR*	2232	GEA00028039	NM_004110.3:1123	Hs01031618_g1
*GDAP2*	54834	GEA00028183	NM_001135589.1:640	Hs00214424_m1
*GDF11*	10220	GEA00006411	NM_005811.3:3590	Hs00195156_m1
*GNL3L*	54552	GEA00029786	NM_001184819.1:1935	Hs00535521_m1
*HIST1H3J*	8356	GEA00014232	NM_003535.2:364	Hs00361917_s1
*HMGCS1*	3157	GEA00026981	NM_002130.4:420	Hs00940429_m1
*HNRNPL*	3191	GEA00029122	NM_001533.2:757	Hs00704853_s1
*LY96*	23643	GEP00055624	NM_015364.2:360	Hs01026734_m1
*MAPK13*	5603	GEP00055633	NM_002754.3:1050	Hs00559623_m1
*MTR*	4548	GEA00030234	NM_000254.2:6816	Hs00299285_s1
*NQO1*	1718	GEA00013124	NM_000903.2:790	Hs02512143_s1
*OR5B21*	219968	GEA00014658	NM_001005218.1:292	Hs02339238_s1
*PFAS*	5198	GEA00032277	NM_012393.2:4655	Hs00389822_m1
*PHLDA3*	23612	GEA00015249	NM_012396.3:532	Hs01926548_s1
*RFC2*	5982	GEP00055631	NM_181471.1:835	Hs00945948_m1
*SFPQ*	6421	GEP00055626	NM_005066.2:1995	Hs00192574_m1
*SLC37A4*	2542	GEP00055634	NM_001164277.1:1248	Hs00184616_m1
*SQLE*	6713	GEP00055620	NM_003129.3:250	Hs01123768_m1
*TLR6*	10333	GEA00012581	NM_006068.2:2530	Hs00271977_s1
*TMEM97*	27346	GEP00055627	NM_014573.2:2055	Hs00299877_m1
*TXNRD1*	7296	GEA00013820	NM_001093771.1:1009	Hs00917067_m1
*ABCB4*	5244	GEP00055630	NM_018849.2:2125	Hs00240956_m1
*GAPDH*	2597	GEP00055153	NM_002046.3:972	Hs03929097_g1
*HPRT1*	3251	GEP00055483	NM_000194.1:240	Hs99999909_m1

A set of 30 transcripts from the GPS was analyzed on all three platforms. Transcripts are listed by gene symbol, Entrez Gene ID, DELTAGene™ assay ID, NanoString® probe ID and Life Technologies™ Assay ID. In addition to transcripts from the GPS, three reference transcripts, *ABCB4, GAPDH* and *HPRT1* were analyzed on the evaluated platforms and used for normalization of data.

**Table 4:** **Comparison of platforms**

Platform	Affymetrix®	nCounter®	BioMark HD™	OpenArray®
Technology	Microarray	Hybridization	RT-qPCR	RT-qPCR
Input material	cDNA	Total RNA	Pre-Amp cDNA	cDNA
Sample consumption	300 ng total RNA/sample	200 ng total RNA/sample	125 ng cDNA/sample	120 ng cDNA/sample
Time for analysis	~4 days from RNA to results	22 h from RNA to results with a hands on time of 15 minutes.	7 h from cDNA to results with a hands on time of ~ 4 h	3 h from cDNA to results with a hands on time of 20 minutes.
Protocols	Difficult	Easy	Medium	Easy
Price^a^	$$$$	$$$	$	$$

Comparison are based on the 48 samples analyzed in the present study. ^a^ Platforms were graded based on total cost to analyze the complete set of 48 samples. The number of $ denotes the relation in costs between the platforms, with the highest number assigned to the most expensive platform.

### Preprocessing of data

Gene expression data obtained on evaluated platforms were normalized prior to any analysis as described in Methods. Due to issues with a batch of samples during RNA preparation, one of the replicates from the following chemical stimulations had to be removed from analysis on all platforms: p-phenylendiamine, geraniol, eugenol and 1-butanol. In addition, one of the replicates from the following chemical stimulations had to be removed from analysis on nCounter® platform due to platform specific issues: 2-aminophenol and water. Total dataset analyzed on nCounter® consisted of 42 samples. In addition to the samples removed due to issues during RNA preparation, one of the replicates from the following chemical stimulations had to be removed from analysis on BioMark HD™ and OpenArray® platforms due to insufficient amounts of cDNA: propylene glycol, methyl salicylate, geraniol and dimethyl sulfoxide. Total dataset analyzed on BioMark HD™ and OpenArray® consisted of 40 samples. All stimulations except geraniol was present in at least biological duplicates. For chemical stimulations consisting of two replicates, averaged values were calculated from duplicate reactions instead of on triplicate as applied to remaining stimulations. The total number of sample shared between all platforms comprised 38 samples.

### Detection sensitivity

To determine how evaluated platforms compared in sensitivity to each other, we estimated the total number of transcripts detected on each platform. Using the set of 38 chemical stimulations shared between all platforms and measuring the expression levels of all 33 transcripts in each stimulation, a total of 1254 transcripts were assayed on each platform. Transcripts were defined as either detected or undetected in a certain chemical stimulation based on platform specific criteria as described in Methods. The overall number of detected transcripts in all samples was higher on the BioMark HD™ platform (1246/1254) in comparison to both the OpenArray® platform (1210/1254) and the nCounter® platform (1128/1254). In order to generate a uniform set of transcripts for comparative analysis between the platforms, we chose to include only those transcripts detected in at least two of the three replicate reactions for all stimulations and on all of the evaluated platforms during downstream analysis. Identities and number of detected transcripts for all stimulations and on each platform are summarized in Table 5. While all transcripts were detected in at least two of the three replicates for all stimulations on BioMark HD™, one transcript (*ABCB4*) on the OpenArray® platform, and three transcripts (*ABCB4, ORB5B21 and ADAM20*) on the nCounter® platform, did not fulfill the stringent requirements for use in downstream analysis. Subsequently, *ABCB4, ORB5B21* and *ADAM20* were removed from the set of transcripts. Two of the removed transcripts, *ORB5B21* and *ADAM20* originated from the GPS while the transcript *ABCB4* was originally considered to be used as a reference gene. All of the removed transcripts were confirmed as low expressed transcripts in the MUTZ-3 cell line based on microarray data (data not shown). The total gene set used for further analysis thus consisted of a total of 28 transcripts from the GPS.

**Table 5:** **Comparison of the number of detected genes in the 38 samples shared between all platforms**

Gene symbol	nCounter®	BioMark HD™	OpenArray®
*ABCB4*	1 (3%)	31 (82%)	18 (47%)
*FDXR*	D	37 (97%)	36 (95%)
*OR5B21*	0 (0%)	D	37 (97%)
*ADAM20*	11 (29%)	D	35 (92%)
*HIST1H3J*	36 (95%)	D	36 (95%)
*GDF11*	30 (79%)	D	D
*LY96*	D	D	36 (95%)
*TLR6*	36 (95%)	D	D
*CD86*	33 (87%)	D	36 (95%)
*FASN*	D	D	37 (97%)
*PFAS*	D	D	36 (95%)
*MAPK13*	37 (97%)	D	D
*GDAP2*	D	D	36 (95%)
*TMEM97*	35 (92%)	D	D
*DHX33*	35 (92%)	D	D
*RFC2*	D	D	37 (97%)
*ACLY*	D	D	35 (92%)
*TXNRD1*	D	D	37 (97%)
*HPRT1*	D	D	37 (97%)
*GAPDH*	D	D	37 (97%)

The set of 33 transcripts were measured in the 38 chemical stimulations shared between all platforms. The table illustrates the number of detected calls for a specific transcript across the 38 samples with percentage in parentheses. Table lists only transcripts declared as undetected in any of the stimulations on either nCounter®, BioMark HD™ or OpenArray®. D denotes that transcript is present in all samples.

### Intra-platform reproducibility and data consistency

**Figure 3:**
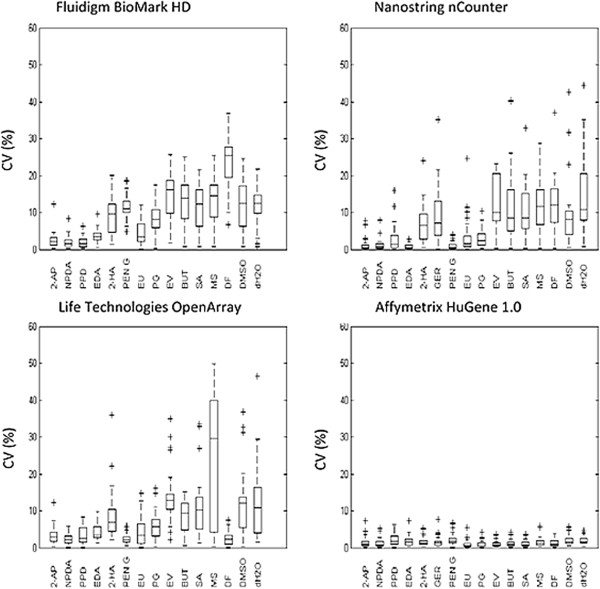
**Distribution of replicate coefficient of variation (CV).** As a measure of intra-platform precision and data consistency, the replicate CV of 28 transcripts present in all stimulations were determined from triplicate stimulations and illustrated in a series of box and whiskers plots. The interquartile range (25^th^ and 75^th^ percentile), median (Gap) and the 10^th^ and 90^th^ percentile values as well as outliers are indicated in each plot.

We chose to evaluate precision and to measure data consistency within each platform by calculating coefficient of variation (CV%) between biological replicate measurements. Calculations of CV were performed on intra-platform replicates for each chemical stimulation using quantitative gene expression signals from the subset of 28 transcripts shared between the evaluated platforms. The distribution of replicate CV values across all transcripts are summarized in a series of box and whiskers plots visualized in Figure 3. All platforms demonstrated good reproducibility of gene expression data. The replicate CV median values for Affymetrix® were the lowest amongst the platforms, ranging from 1-3% for all chemical stimulations. The replicate CV median values for the majority of the chemical stimulations measured on nCounter®, BioMark HD™ and OpenArray® platforms were in the range of 5-15%, although the distribution of replicate CV varied slightly between various chemical stimulations measured on the same platform. Examples include larger dispersion of replicate CV values for the stimulations ethyl vanillin, 1-butanol, salicylic acid, methyl salicylate, dimethyl formamide, dimethyl sulfoxide and water in comparison to 2-aminophenol, 2-nitro-1,4-phenylendiamine, p-phenylendiamine and ethylendiamine. However, the pattern of dispersion of the CV values for the different stimulations seems to be comparable across the evaluated platforms.

### Inter-platform consistency

**Figure 4:**
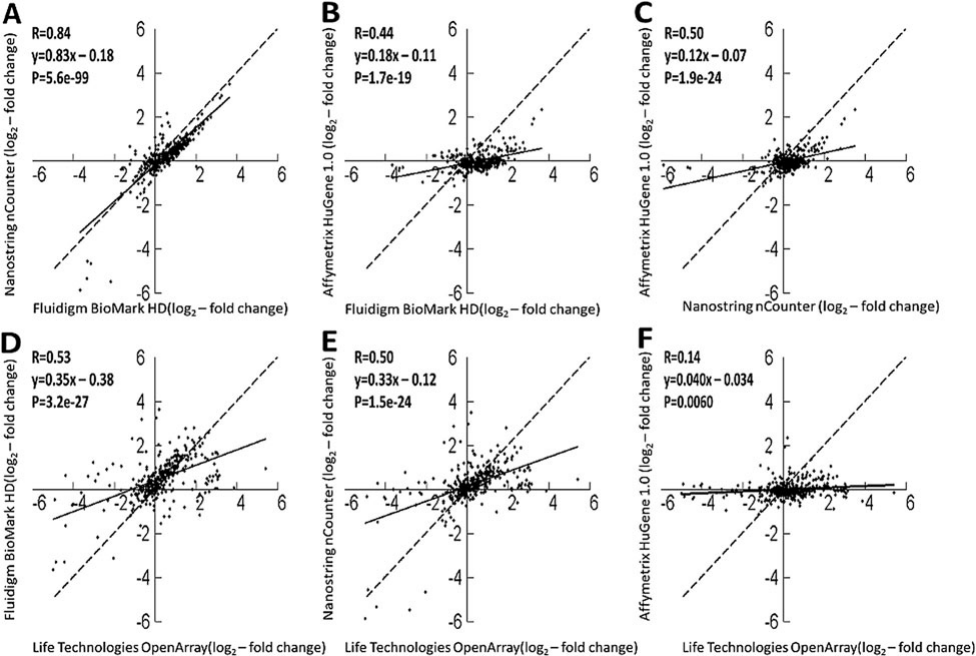
**Fold change correlations between gene expression data quantified by the various platforms.** Cross-platform comparisons were performed by evaluating the concordance of log_2_ fold changes by pair wise regression analysis of fold differences for chemical stimulations in comparison to vehicle controls. The average log_2_ fold changes for 28 transcripts present in all stimulations and detected on all three platforms were subjected to bivariate analysis. Subplots **(A-F)** illustrates the correlation between the following platforms: **A)** nCounter® and BioMark HD™, **B)** Affymetrix® and BioMark HD™, **C)** Affymetrix® and nCounter®, **D)** BioMark HD™ and OpenArray®, **E)** nCounter® and OpenArray®, **F)** Affymetrix® and OpenArray®. The solid line in each plot illustrates the linear regression fit while the dashed line represents the 1.0 slope of complete concordance. Pearson correlation coefficients (R) and equation for the linear fit (y) are indicated for each subplot. P-values for hypothesis of no correlation were calculated using Fisher’s transformation (P).

We further evaluated reliability of gene expression data measured on the nCounter®, BioMark HD™ and OpenArray® platforms by comparing their abilities to measure transcripts in the GARD signature to each other and to Affymetrix® microarrays. Absolute signals of gene expression values generated on the different platforms could not be compared directly since the different platforms utilized different chemistry, quantitation, and normalization methods. Therefore, we evaluated the correlation between data in fold change matrices. Cross-platform comparisons were performed by evaluating concordances of log_2_ fold changes by pair wise regression analysis of fold differences for chemical stimulations in comparison to vehicle controls. Biological replicate measurements for the 38 samples shared between all platforms were used to calculate the average log_2_ fold changes across the 28 transcripts present in each stimulation. Dimethyl sulfoxide and water were used as reference samples. The chemical stimulation geraniol was removed from the analysis as no replicate samples were available. This left a dataset consisting of 364 matched measurement pairs (averaged values from 13 stimulations). The matched measurement pairs from each platform were pair-wise plotted against each other and subjected to bivariate analysis. Pearson correlation coefficients and linear fits to the log_2_ ratios were calculated for each pair-wise platform comparison (Figure 4). Strong correlation was observed between log_2_ fold change measurements on nCounter® and BioMark HD™ platforms (Figure 4A, R = 0.84). Lower correlations were observed of either of BioMark HD™ (Figure 4B, R = 0.44) or nCounter® (Figure 4C, R = 0.50) to Affymetrix®. Low correlations were also observed between log_2_ fold change measurements of either of BioMark HD™ (Figure 4D, R = 0.53), nCounter® (Figure 4E, R = 0.50) and Affymetrix® (Figure 4F, R = 0.14) with the OpenArray® data. Level of compression or expansion of gene expression data measured on the different platforms were evaluated by comparing the slope of the best fitted line of a least square linear regression of the log_2_ fold changes between pairs of platforms to the ideal slope of 1. Only a minor compression was observed between nCounter® and BioMark HD™ (slope: 0.83). Compression effects were observed for both nCounter® (slope = 0.33) and BioMark HD™ (slope 0.35) in relation to OpenArray®. Affymetrix® data compressed log_2_ fold change measurements relative to the other platforms (slope 0.12, 0.18, 0.040 for BioMark HD, nCounter® and OpenArray® respectively).

### Comparison of transcriptional profiles from different platforms as predictive models for skin sensitization

**Figure 5:**
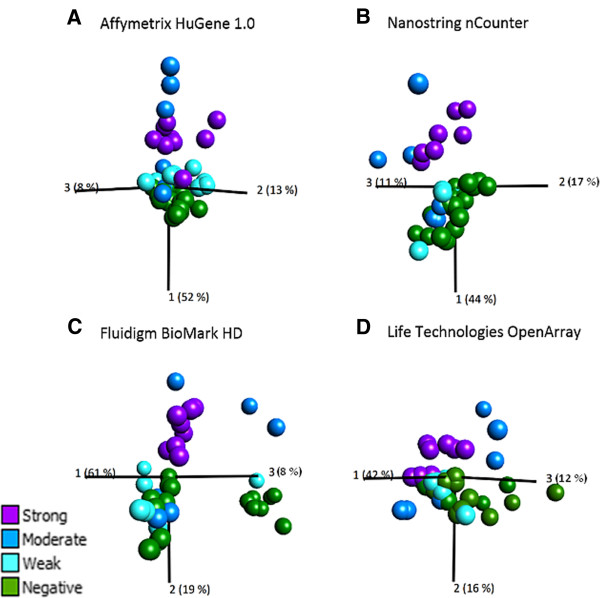
**Principal component analysis (PCA) of gene expression data after 24 h of chemical stimulation.** To investigate overall similarities of gene expression data and to outline relevance of data from evaluated platforms for the GARD assay, gene expression data from 28 transcripts shared between all platforms were used for construction of PCA plots in the software Qlucore. Samples were colored according to sensitizing potency as defined by the LLNA assay. Subplots (A-D) illustrates PCA plots constructed from gene expression data obtained on the various platforms. **A)** Affymetrix®, 48 stimulations (weak (n = 9), moderate (n = 6), strong (n = 9), non-sensitizer (n = 24)). **B)** nCounter®, 42 stimulations (weak (n = 7), moderate (n = 6), strong (n = 7), non-sensitizer (n = 22)). **C)** BioMark HD, 39 stimulations (weak (n = 6), moderate (n = 6), strong (n = 8), non-sensitizer (n = 19). **D)** OpenArray®, 36 stimulations (weak (n = 6), moderate (n = 6), strong (n = 8), non-sensitizer (n = 16).

**Figure 6:**
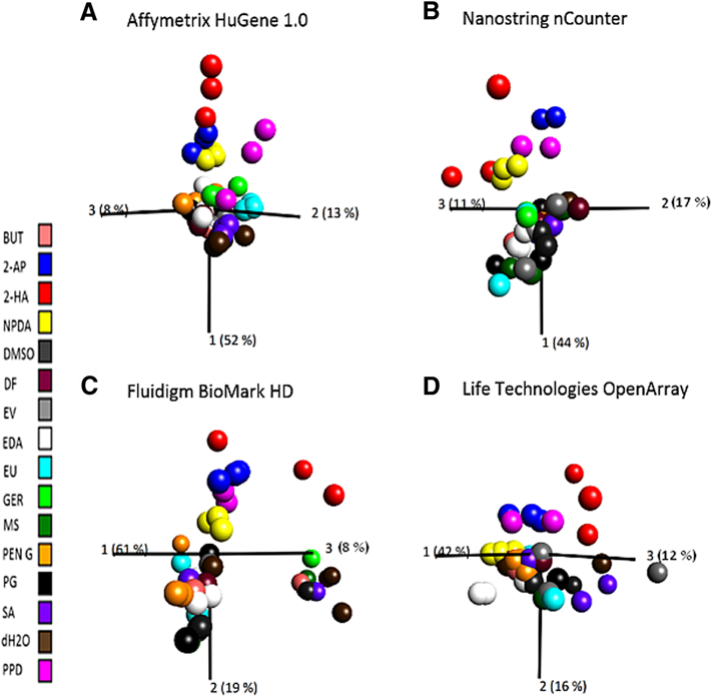
**Principal component analysis (PCA) of gene expression data after 24 h of chemical stimulation.** To investigate consistency of gene expression data from evaluated platforms, samples from Figure 5 were instead colored according to chemical stimulation. Subplots **(A-D)** illustrates PCA plots constructed from gene expression data obtained on the various platforms. **A)** Affymetrix®, 48 stimulations. **B)** nCounter®, 42 stimulations. **C)** BioMark HD, 39 stimulations. **D)** OpenArray®, 36 stimulations.

We evaluated overall structure and relevance of gene expression data measured on evaluated high-throughput platforms on a per application basis in order to determine if platforms could be used to distinguish sensitizers from non-sensitizers in the GARD assay. Principal component analysis (PCA) was used to visualize gene expression data from the various platforms. PCA plots were constructed in the software Qlucore Omics Explorer v.2.1 using the subset of 28 transcripts shared between evaluated platforms as variable input. Construction of PCA plots based on the 40 samples in the BioMark HD™ and OpenArray® sample datasets revealed significant outliers that had to be removed, otherwise they would have prevented visualization and classification of separate groups (data not shown). From the BioMark HD™ data set, one of the replicate of dimethyl formamide had to be removed, leaving a dataset comprising 39 samples. From the OpenArray® data set, one replicate of dimethyl formamide, 1-butanol, methyl salicylate and water had to be removed, leaving a dataset comprising 36 samples. All 42 samples measured on nCounter® were employed for construction of the nCounter® PCA plot. For comparative reasons, a PCA plot was constructed based on Affymetrix® gene expression measurements of the 48 samples originally selected for this evaluation using the subset of 28 transcripts shared between evaluated platforms as variable input. To determine if gene expression measurements from evaluated platforms could be used to distinguish sensitizers from non-sensitizers, each sample in the PCA plots was colored according to sensitizing potency as defined by the LLNA assay (Figure 5). Neither of the evaluated platforms were able to achieve a complete separation of sensitizers and non-sensitizers. The PCA plot constructed from nCounter® data showed highest level of similarity to the Affymetrix® PCA plot. For both these platforms, discrimination between sensitizers and non-sensitizers were seen along the first principal component. While all strong sensitizers clustered together, separated from the non-sensitizers, a few of the moderate and all the weak sensitizers clustered close to the non-sensitizers for the Affymetrix® platform, and together with the non-sensitizers for the nCounter® platform. The separation profiles for both the BioMark HD™ and the OpenArray® platforms demonstrated a clear discrimination between strong sensitizers and non-sensitizers while a few moderate sensitizers and most weak sensitizers clustered together with non-sensitizers. However, in contrast to the nCounter® and the Affymetrix® data, discrimination between sensitizers and non-sensitizers was observed along the second principal component. To further investigate the quality and similarity of gene expression data produced on the various platforms for the different chemical stimulations, each sample in the PCA plot was instead colored according to stimulating agent (Figure 6). Two important observations were made: Firstly, the moderate sensitizer that consistently clustered together with the non-sensitizers for all platforms could be identified as being the same chemical, ethylendiamine. Secondly, replicate stimulations grouped together in all PCA plots, indicating high quality data from all platforms.

## Discussion

The GARD assay was recently developed as an *in vitro* alternative to animal testing for identification and classification of skin sensitizers. The assay classifies chemicals as either skin sensitizers or non-sensitizers with high accuracy, sensitivity and specificity, using a genomic biomarker signature in the myeloid cell line MUTZ-3. The MUTZ-3 cell line is ideal for assay development as they resemble dendritic cells (DCs) which are important modulators of immune reactions in response to foreign substances [24]. The use of a cell line in the GARD assay ensures a constant supply of cells and enables a large number of cells to be grown via standardized protocols in short time, allowing for a capability to scale up the format of the assay. We believe that the testing strategy behind the GARD assay has potential to replace animal experimentation for classification of chemicals, but that the technical platform currently used for transcriptome analysis, the microarray platform, is a limiting factor for adaption of GARD into a routine assay for analysis and screening of potential sensitizers. To improve GARD and place the assay in a better position for formal validation following an established assessment procedure with the ultimate aims to simplify assay procedures, improve technical parameters and increase sample throughput, we assessed the performance of three high throughput platforms for gene expression measurements and correlated their performance metrics against our previously generated microarray data [10]. We measured the levels of 30 representative genes from the GPS across 48 samples on all platforms. Samples comprised 16 chemical stimulations in biological triplicate reactions and included both sensitizers and non-sensitizers. To achieve a comprehensive evaluation, we applied an experimental design allowing us to perform measurements on all platforms from the same chemical stimulation and a single RNA preparation. Thus, only platform-specific protocols downstream from RNA isolation discriminated between measurements on the different platforms.

The key findings from the current study are threefold. Firstly, good intra-platform reproducibility of gene expression data for all platforms was observed. Secondly, all evaluated platforms displayed modest to low correlation of gene expression measurements compared to Affymetrix® microarray data. Thirdly, all platforms were able to discriminate most of the extreme and moderate skin sensitizers from non-sensitizers when gene expression data obtained from the various platforms were subjected to PCA analysis.

High intra-platform reproducibility of gene expression measurements was considered a key criterion during the evaluation of platforms. High reproducibility might reduce number of replicates needed for measurements, affecting experimental design, sample throughput and eventually also costs. Considering that evaluated platforms not only use different technology for gene expression measurements but also different protocols for preparation of samples prior to final quantification, we chose to calculate precision based on biological replicate measurements where replicates for each stimulation was prepared individually for analysis according to protocols for a certain platform. Thus, the estimated precision included both technical variations during final measurements as well as variations possibly introduced during sample preparation prior to quantification. The present study demonstrates high intra-platform precision for all platforms. The majority of replicate CV median measurements for nCounter®, BioMark HD™ and OpenArray® were in the range between 5-15% which was slightly higher than the CV values observed on the Affymetrix® platform. In contrast to the CV values observed on the Affymetrix® platform, the distribution of replicate CV values varies between stimulations on evaluated platforms. The distribution of replicate CV values for a certain stimulation is however comparable across the evaluated platforms. Considering that a single stimulation was prepared for analysis on nCounter®, BioMark HD™ and OpenArray®, while Affymetrix® data was based on previously generated data it is likely that the higher CV values observed for the evaluated platforms in comparison to Affymetrix® originate from sample preparation and not from the quantification itself. In addition, evaluated genes were chosen by a data-driven approach, using the Affymetrix® data as an exploratory data set. Thus, the very low CV values observed for Affymetrix® data might be explained by a selection bias. The multiple steps included in sample preparation (RNA to cDNA preparation, data acquisition, normalization of data) are all possible steps for introduction of variations. The nCounter® system has the simplest protocol since measurements can be performed directly on RNA samples and in a single reaction. The OpenArray® system requires reverse transcription, but most steps in the analysis is fully automated using a mechanic liquid handler. Finally, the BioMark HD™ system requires both reverse transcription, pre-amplification and several steps of technical pipetting. However, these differences did not seem to affect the reproducibility of the measurement to any large extent as indicated by the similarity in CV values across platforms.

In contrast to reproducibility measurements, we also reported differences in the number of transcripts that could be detected on each of the evaluated platforms. Reasons for not detecting a transcript can be explained by a bad probe for the specific gene or insufficient sample target concentration. Since high frequencies of undetected genes correlated well with low levels of expression, we suggest that the major contribution in this study was due to transcript levels under the limit of detection for the specific platform. Results indicate superior sensitivity of the BioMark HD™ platform in comparison to both nCounter® and OpenArray®. The increased detection sensitivity may be linked to the additional cDNA pre-amplification step implemented prior to RT-qPCR on BioMark HD™. In contrast, the nCounter® platform does not involve any target amplification and the OpenArray® technology involves amplification only during the RT-qPCR reaction when using standard protocols. An unexpected observation was also that the performance of the reference transcripts were different between the platforms (data not shown). While both *GAPDH* and *HPRT1* performed equally well on nCounter®, only *GAPDH* performed satisfactory on the BioMark HD™ and the OpenArray® platforms suggesting that care must be taken in selecting appropriate reference genes.

Equally important as reproducibility was to achieve consistency of gene expression measurements across platforms. This would provide a possibility to directly compare new measurements to previous data and to use SVM model trained on historical microarray data for classification of new samples measured on alternative platforms. However, the cross-platform comparison of log_2_ fold change measurement indicates modest to poor correlation between gene expressions data measured on the different platforms. We suggest that the major contributions to poor correlations are attributable to either platform specific differences in protocols, data processing or differences inherent to each technology. Contributing factors may include slight differences in probe sequences, small differences in the location of probes or primers and the lack of industrial standards across different technologies. Since the selection of primers and probes in this study was based on probe annotations, slight differences in the location of probes or primers on the target sequence might result in the detection of different types of splice variants on the different technologies. Previous experiments have shown that it is possible to achieve improved correlations across platforms using probes matched at exon levels [25]. Other groups have also stated that differences in probes across microarray platforms can be a major cause of discordance [26, 27]. We do not have full access to the complete primer and probe sequences for all platforms preventing us from investigating the actual contribution of this factor in this experiment. We feel that the low concordance of gene expression measurements between evaluated platforms and to Affymetrix® microarray data will prevent a direct comparison of previously generated microarray data to new measurements on an alternative platform in this type of application.

Finally, we evaluated the overall structure and similarities of gene expression measurements from the various platforms in order to investigate if data from evaluated platforms contained enough biological information to distinguish sensitizers from non-sensitizers in PCA analysis. All platforms were able to distinguish strong sensitizers as well as several of the moderate sensitizers from the non-sensitizers. However, information contained in the gene expression data were not sufficient to distinguish weak sensitizers from non-sensitizers on any of the evaluated platforms. Previous work has reported variations in transcriptional profiles related to the relative potency of stimulating agent [10], indicating a smaller variation between weak sensitizers and non-sensitizers in comparison to strong and moderate sensitizers. Although significant efforts were made in order to select target genes with highest discriminatory power between sensitizers and non-sensitizers in this study, the inability to accurately classify weak sensitizers on the evaluated platforms could potentially be an artifact generated by the smaller number of genes included in this study (n =28) in comparison to the complete GARD signature (n = 200). Therefore, we filtered the Affymetrix® data set to include the same genes as analyzed by evaluated platforms. Results indicate that the genes selected for this study contains sufficient information to distinguish also the weak sensitizers as the Affymetrix® data illustrated a more obvious separation between weak sensitizers and non-sensitizers in comparison to evaluated platforms (Figure 5). Considering that the GARD signature was initially selected based on Affymetrix® microarray data and the poor correlations observed between Affymetrix® and evaluated platforms, we suggested that a reasonable explanation to why evaluated platforms were not able to completely distinguish all sensitizers from all non-sensitizers derives from differences in technology between platforms, i.e., the same factors that contributed to the poor correlation between platforms as discussed above. In support of this argument, the PCA plot constructed from data measured on the hybridization based nCounter® platform shows most similarities to the PCA plot constructed from Affymetrix® data. Alternatively, the observed differentiation between weak sensitizers and non-sensitizers in the Affymetrix® data may also be due to a selection bias, as the Affymetrix® data is the historical data set used to identify the genomic predictors. Therefore, we cannot exclude the possibility that including a larger set of genes from the GPS to be analyzed on the evaluated platforms might provide more information relevant for the separation also of weak sensitizer as a larger set of genes would allow for a data driven approach to select for better predictors also on evaluated platforms. In addition, we observed that the separation of sensitizers were seen along the second principal component in PCA plots for gene expression data from the BioMark HD™ and OpenArray® data instead of along the first principal component as seen with Affymetrix® and nCounter® data. The observed separation along the second principal component indicates that much of the variation in the datasets may be attributable to systematic artifacts from the technologies and not from the transcriptional variations. This has previously been shown for other high-throughput technologies as reviewed by [28].

Other important parameters to consider are price per assay, simplicity of protocols, time to results and sample throughput. These parameters had to be balanced in relation to the technical parameters in order to select a suitable platform for GARD. In comparison to the Affymetrix® platform, evaluated platforms enable higher sample throughput, having shorter turnaround time for results and provides more flexible solutions for focused gene expression of a specific sets of genes. The nCounter® platform has the additional advantage of not requiring reverse transcription, being highly multiplexed regarding the amount of transcripts to be measured in a single reaction, requires low amount of hands-on time, provides fully automated liquid handling steps and has the easiest workflow. The BioMark HD™ system is most cost effective and provides a straightforward and simple method for data normalization. The OpenArray® system has an easy work flow and streamlined protocol were most liquid handling steps are automated and enables progression from cDNA into results in less than three hours.

In summary, all of the evaluated platforms produced high quality gene expression data, demonstrated acceptable reproducibility of gene expression measurements and retained discriminatory power for separation of strong and moderate sensitizers. Although slight differences between the high-throughput platforms in terms of cost per sample, sample throughput and simplicity of protocols were observed, the evaluated platforms were superior in relation to the microarrays when considering these parameters.

## Conclusions

In this study, we evaluated the performance of three high-throughput platforms using a restricted set of probes from the GARD biomarker signature. We showed that it was possible to achieve acceptable detection sensitivity, reproducibility and discriminatory power between strong and moderate sensitizers and non-sensitizers in the GARD assay and at the same time reduce time to results and assay costs, simplify assay procedures and increase sample throughput by using a high throughput platform. As a final remark, we conclude that all of the evaluated platforms are suitable candidates to replace microarrays as the technical platform for GARD. Changing the platform for gene expression measurement will be a first step in order to prepare GARD for validation and industrial screening of potential sensitizers.

## Methods

### Chemicals

A panel of 16 chemical compounds comprising eight sensitizers and eight non-sensitizers as defined by the LLNA assay were used for stimulation of cells. The sensitizers were 2-aminophenol, 2-nitro-1,4-phenylenediamine, 2-hydroxyetylacrylate, p-phenylenediamine, etylenediamine, geraniol, penicillin G and eugenol. The non-sensitizers were propylene glycol, ethyl vanillin, 1-butanol, salicylic acid, methyl Salicylate, dimethyl formamide and the vehicle controls dimethyl sulfoxide and water. All chemicals were purchased from Sigma-Aldrich (St. Louis, MO). Chemical compounds were dissolved in either dimethyl sulfoxide or water [10]. Each chemical compound was diluted into a concentration corresponding to the GARD input concentration. Establishment of GARD input concentration has been extensively described elsewhere [10, 29]. Sensitizing potency, GARD input concentration and solvents for each chemical compound are summarized in Table 2.

### Cell cultures, phenotypic analysis and chemical stimulations

Maintenance, phenotypic analysis and stimulation of the human myeloid leukemia-derived cell line MUTZ-3 were performed as previously described [10]. In short, phenotypic analysis of MUTZ-3 cells was performed using flow cytometry prior to each experiment to ensure that cells were not differentiated. Monoclonal antibodies used during phenotypic analysis: CD1a (DakoCytomation, Glostrup, Denmark), CD34, CD86, HLA-DR (BD Biosciences, Franklin Lakes, NJ), all FITC-conjugated. CD14 (DakoCytomation), CD54, CD80 (BD Biosciences), all PE-conjugated. Propidium iodide (BD Biosciences) was used to estimate cell viability and FITC- and PE- conjugated mouse IgG1 (BD Biosciences) were used as isotype controls. Samples were analyzed on FACSCanto II instrument using FACS Diva software for data acquisition. A total of 10, 000 events were acquired and data were imported into FCS Express V4 (De Novo Software, Los Angeles, CA) for further analysis. Gates were set to exclude non-viable cells and cell debris based on light scattering properties. During chemical stimulation, MUTZ-3 cells were seeded in 24 well-plates and stimulated with chemical compounds. All stimulations were performed in biological triplicates, performed at different time points using different cell cultures. After 24 h incubation, cells were harvested. Maturity state of the cells were controlled by analyzing cell surface expression of CD86 using flow cytometry as described above. In parallel, harvested cells were lysed in TRIzol® reagent (Life Technologies™, Carlsbad, CA) and stored at −20°C until RNA extraction.

### RNA extraction and cDNA preparation

RNA was isolated from chemically stimulated MUTZ-3 cells using TRIzol® reagent. Purity and concentration of RNA in each sample were confirmed using Agilent Bioanalyzer 2100 (Agilent Technologies, Santa Clara, CA) according to standard protocol provided by the manufacturer. Each sample was diluted into 200 ng/μl of total RNA and divided into two identical 10 μl samples (2 μg total RNA). One of the samples was further diluted into 20 ng/μl of total RNA and analyzed on nCounter® platform. The other sample was reverse transcribed into cDNA using the High capacity cDNA reverse transcription kit (Life Technologies™) according to standard protocols provided by the manufacturer. In short, 10 μl of a reverse transcription master mix containing MultiScribe™ reverse transcriptase and random primers was prepared for each sample and added to 2 μg of total RNA (10 μl at 200 ng/μl) for a 20 μl reaction. The following thermal protocol was used: 25°C, 10 min; 37°C, 120 min; 85°C, 5 min; 4°C hold. A reaction efficiency of 100% was assumed. The cDNA samples were divided into two identical 10 μl samples and stored at −20°C until further downstream processing for analysis on the BioMark HD™ platform and the OpenArray® platform.

### Primers and probes

A total of 33 transcripts were selected for the evaluation. The set of transcripts included 30 genes from GPS as well as three reference genes (*ABCB4, HPRT1, GAPDH*) for normalization of data. A probe set targeting the 33 transcripts was obtained from NanoString® (NanoString® Technologies, Seattle, WA) for analysis on the nCounter® platform. Pairs of primers targeting the same 33 transcripts were obtained from DELTAGene™ (Fluidigm®, San Francisco, CA) for analysis on the BioMark HD™ platform. DELTAGene™ quantification of gene expression was based on SYBR® Green hybridization chemistry. Pairs of primers and probes targeting the same 33 transcripts were obtained from Life Technologies™ (Life Technologies™, Carlsbad, CA) for analysis on the OpenArray® platform. OpenArray® quantification of gene expression was based on TaqMan® hydrolysis chemistry. A complete list of accession numbers for transcripts is provided in Table 3.

### Measurement of gene expression on nCounter® system

Total RNA samples from chemically stimulated MUTZ-3 cells, prepared as described above, were sent to NCCR Frontiers in Genetics (Geneva, Switzerland) on dry ice for analysis on the nCounter® system. An amount of 200 ng (10 μl) of total RNA from each sample was analyzed on the nCounter® system using standardized protocol for gene expression analysis provided by NanoString®. Each sample was analyzed in a separate multiplexed reaction. Data was imported into nSolver™ analysis software using NanoString® raw code count collector tool. Normalization of data was performed according to NanoString® analysis guidelines. For negative control normalization, the mean of the counts for all negative controls in each sample plus two standard deviations was subtracted from counts for the remaining transcripts in same sample. For positive control normalization, a normalization reference was first generated by calculating the sum of the counts for the positive spike in transcripts in each sample and then calculating the average of these sums across all samples. A scaling factor for each sample was then calculated by dividing the sum of the counts from the positive spike in transcripts from each sample with the normalization reference. Remaining code counts were multiplied by the sample specific scaling factor. Normalized counts were compiled into Microsoft Excel and further normalized against the reference genes *GAPDH* and *HPRT1*. For normalization to reference genes, the geometric mean of the counts for *GAPDH* and *HPRT1* for each sample was calculated, and the average across all samples was used as the normalization reference. A scaling factor for normalization of remaining code counts was calculated and applied as described above for positive control normalization. Reference genes were selected based on internal control parameters included in an nSolver Excel macro developed by NCCR Frontiers in Genetics using the geNorm method [30]. For a gene to be considered as detected, the normalized gene counts for a specific transcript had to be significantly (Student’s t test, p < 0.05) above background threshold count value for the negative controls.

### Measurement of gene expression on BioMark™ HD system

Specific Target Amplification (STA) was performed on cDNA samples using TaqMan® PreAmp Master Mix (Life Technologies™) and standard protocols provided by manufacturer. In short, 1.25 μl of each cDNA sample was combined with 2.5 μl of 2x TaqMan PreAmp Master Mix in wells of a 96-well PCR plate. 0.5 μl of a pre-amplification primer mix comprising a mixture of pooled DELTAGene™ assays (500nM of each primer) was mixed with 0.75 μl dH_2_O and added to each sample. The PCR plate was transferred to a thermal cycler and subjected to the following thermal protocol: 95°C, 10 min; 14 cycles (95°C ,15 s; 60°C, 4 min); 4°C hold. Samples were treated with Exonuclease I (New England BioLabs, Ipswich, MA) to remove unincorporated primers by adding 2 μl (at 4 U/μl) to each STA reaction. The PCR plate was once again transferred to a thermal cycler and subjected to the following thermal protocol: 37°C, 30 min; 80°C, 15 min; 4°C hold. All samples were then diluted 10× in TE-buffer (10 mM Tris–HCl, 0.1 mM EDTA, pH 8) (Sigma-Aldrich) and sent to Veterinærinstituttet (DTU, Copenhagen, Denmark) on dry ice for analysis on the BioMark HD™ system. Operation of instruments and handling and processing of the IFC controller HX were performed using standardized protocol for gene expression analysis provided by Fluidigm®. Samples were prepared for loading on a 96:96 Dynamic array™ IFC by combining 2.25 μl of each preamplified cDNA sample with 2.5 μl 2× SsoFast™ EvaGreen® Supermix with low ROX (BioRad, Hercules, CA) and 0.25 μl 20× DNA binding dye loading reagent (Fluidigm®). A total of 5 μl of each pre amplified cDNA sample and 5 μl of each DeltaGene assay were dispensed into their corresponding inlets on the Dynamic array™ and samples were subjected to RT-qPCR reaction using the following thermal cycling protocol: 70°C, 40 min; 60°C, 30 s; 95°C, 1 min; 35 cycles (96°C, 5 s; 60°C, 20 s) and melting curve using a ramp from 60°C to 95°C at 1°C/3 s. Data was collected with Fluidigm® Real-Time PCR analysis software using linear baseline correction method and global auto Cq threshold method. Normalizations of data were performed using comparative Cq method [31]. The reference gene used in this study, *GAPDH*, was selected based on internal control parameters included in software. A transcript was considered as undetected in a certain chemical stimulation when unprocessed Cq value exceeded 35.

### Measurement of gene expression on OpenArray® system

cDNA samples from chemically stimulated MUTZ-3 cells, prepared as described above, were sent to Life Technologies™ demo laboratory (Saint Aubin, France) on dry ice for analysis on the OpenArray® system. Loading of samples, operation of instruments and RT-qPCR cycling were performed using standardized protocol for gene expression analysis provided by Life Technologies™. In short, 1.2 μl of each cDNA sample was combined with 2.5 μl of 2× TaqMan® OpenArray® Real-Time PCR Master Mix (Life Technologies™) and 1.3 μl dH_2_O and transferred to an OpenArray® 384-Well Sample Plate. Samples were loaded into the custom designed OpenArray® plates using QuantStudio™ 12 K flex AccuFill™ system. The OpenArray® plates were removed from the AccuFill™ system, transferred to an OpenArray® carrier, covered with immersion fluid (All reagents from QuantStudio™ 12 K flex OpenArray® accessories kit, Life Technologies™) and loaded into the QuantStudio™ 12 K flex instrument for RT-qPCR cycling. Samples were subjected to standard thermal cycling protocol provided by Life technologies™. Data was collected with Life Technologies™ Expression Suit analysis software v 1.0.3 using linear baseline correction method and the global auto Cq threshold method. Normalization of data was performed using the comparative Cq method [31]. The reference gene used in this study, *GAPDH* was selected based on internal control parameters included in software. A transcript was considered as undetected in a certain chemical stimulation when unprocessed Cq value exceeded 35.

### Measurement of gene expression on Affymetrix® HuGene 1.0 ST arrays

Affymetrix® gene expression data used for comparative analyses in this study was obtained from previously published experiments [10]. Maintenance, phenotypic analysis and stimulation of cells were carried out as described above. Preparation of cDNA, hybridization, washing and scanning of Human Gene 1.0 ST Arrays (Affymetrix®, Santa Clara, CA) as well as RMA normalization of data was performed according to standardized protocol provided by the manufacturer (Affymetrix®) and as previously described [10]. Gene expression data was filtered to contain only the 30 genes from the 16 chemical compound stimulations as analyzed by nCounter®, BioMark HD™ and OpenArray®.

### Inter-platform consistency

Inter-platform consistency was measured by pair-wise plotting of averaged log_2_ fold change data of gene expression measurements obtained on the various platforms. Results were evaluated by calculating Pearson correlation coefficient and linear fits to the log_2_ fold change ratios. Expression ratios were calculated as follows:

Where μ_sample_ is the normalized mean for chemically stimulated samples and μ_control_ is the normalized mean for vehicle controls. For PCR-based methods, ΔCq_sample_ is the normalized mean quantification cycle for chemically stimulated samples and ΔCq_control_ is the normalized mean quantification cycle for vehicle control. Calculations accounts for the fact that Cq values obtained from the RT-qPCR methods as well as the RMA normalized Affymetrix® data were already in log-space.

### Comparison of transcriptional profiles

Normalized gene expression measurements from each platform were compiled into the software Qlucore Omics Explorer 2.1 (Qlucore AB, Lund, Sweden). Gene expression data was filtered to include only those genes detected in at least two of the three replicates. Data was investigated and visualized using Principal Component Analysis.
